# Authors’ Reply: Methodological Concerns in AI Medical Education Frameworks

**DOI:** 10.2196/104447

**Published:** 2026-07-06

**Authors:** Juan S Izquierdo-Condoy, Marlon Arias-Intriago, Laura Montero Corrales, Esteban Ortiz-Prado

**Affiliations:** 1 One Health Research Group, Universidad de Las Américas, Via Nayon S/N, Quito, 170124, Ecuador, 593 992561230; 2 Escuela de Comunicación, Universidad Latina de Costa Rica, San José, Costa Rica

**Keywords:** artificial intelligence, medical education, machine learning, adaptive learning systems, future implications

We thank the authors [1] of the letter “Methodological Concerns in AI Medical Education Frameworks” for their careful reading of our paper [2] and their constructive observations. We welcome the opportunity to clarify the scope of our arguments and the intended role of FACETS (form, application, context, instructional mode, technology, SAMR [substitution, augmentation, modification, redefinition]) in responsible artificial intelligence (AI) integration in medical education.

First, we clarify that the paper by Abdelwanis et al [3] was not cited as direct empirical evidence for educational inequities in low- and middle-income countries (LMICs), nor as evidence for AI implementation, access, effectiveness, or educational outcomes in those settings. We recognize that the review by Abdelwanis et al [3] used a bowtie analysis to examine automation bias, biased data, algorithmic opacity, insufficient validation, and the need for human oversight in health care AI. In our manuscript, this reference was used as conceptual support for general risk mechanisms that may compromise safe and equitable AI adoption. We agree that LMIC-specific claims require more directly contextualized evidence.

We also agree that the study by Ong et al [4] was more closely related to AI training in medical education across income settings, as it showed differences between experts from high-income countries and LMICs in prioritizing AI learning outcomes. However, this evidence should also be interpreted precisely. Ong et al [4] provide empirical evidence on expert perceptions and curricular prioritization, not direct evidence on real-world AI use, effectiveness, access, costs, infrastructure, or educational outcomes among medical students in LMICs. This distinction reinforces our central argument: further contextual, multicenter, and implementation-oriented research is needed [5].

Second, regarding FACETS, we believe the letter interprets our proposal beyond its intended scope. FACETS was not proposed as a predictive, psychometric, or safety-validation instrument, nor as a prospectively validated universal standard. Rather, it was proposed as a conceptual and analytical framework derived from a critical synthesis of published AI applications in medical education to help describe, compare, and interpret heterogeneous interventions across educational form, application, context, instructional mode, technology, and degree of pedagogical transformation.

Accordingly, Luordo et al [6] and Holderried et al [7] were used as empirical examples to illustrate the conceptual applicability of FACETS, not as validation studies. Their limitations reinforce, rather than weaken, the rationale for such a framework. In Luordo et al [6], AI-assisted objective structured clinical examination (OSCE) grading showed promising efficiency and concordance but was systematically stricter than expert evaluators, underscoring the importance of item design, contextual interpretation, and human oversight. In Holderried et al [7], high overall concordance coexisted with lower agreement in selected feedback categories, likely related to ambiguity, category overlap, or interpretive differences. These findings highlight the need for multidimensional frameworks that make explicit the educational purpose, task, context, technological configuration, instructor role, and requirements for local validation.

We appreciate the authors’ effort to enrich the scientific literature on AI in medical education, a field that remains at an early stage. At the same time, we believe that our proposal should be interpreted within the conceptual scope in which it was presented. This discussion reaffirms that our review offers a legitimate and useful conceptual proposal to organize evidence, identify gaps, guide future research, and promote AI adoption in medical education in a pedagogically coherent, ethically responsible, and context-sensitive manner.

## Abbreviations

AI: artificial intelligence
FACETS: form, application, context, instructional mode, technology, SAMR [substitution, augmentation, modification, redefinition]
LMIC: low- and middle-income country
OSCE: objective structured clinical examination
